# 3D genome folding in epigenetic regulation and cellular memory

**DOI:** 10.1016/j.tcb.2025.03.001

**Published:** 2025-04-10

**Authors:** Flora Paldi, Giacomo Cavalli

**Affiliations:** 1 Institute of Human Genetics, CNRS and University of Montpellier, Montpellier, France

## Abstract

The 3D folding of the genome is tightly linked to its epigenetic state which maintains gene expression programmes. Although the relationship between gene expression and genome organisation is highly context dependent, 3D genome organisation is emerging as a novel epigenetic layer to reinforce and stabilise transcriptional states. Whether regulatory information carried in genome folding could be transmitted through mitosis is an area of active investigation. In this review, we discuss the relationship between epigenetic state and nuclear organisation, as well as the interplay between transcriptional regulation and epigenetic genome folding. We also consider the architectural remodelling of nuclei as cells enter and exit mitosis, and evaluate the potential of the 3D genome to contribute to cellular memory.

## Folding principles of the mammalian genome

The earliest observations that implicated chromosome organisation in cellular function were made almost 150 years ago and described the restructuring and segregation of nuclear material during cell division [1]. The idea that chromosome structure had a role in interphase nuclei and could modulate cellular processes such as gene expression, emerged more than hundred years later during the 1980s [2], with the first definitive evidence of *in vivo* regulatory contacts dating to 2002 [3,4]. The recent explosion of sequencing-based and imaging techniques [5] has led to a deeper understanding of the 3D folding of the genome [6] and the mechanisms that drive its multilayered organisation. Two major modes of folding have emerged that operate in mammalian genomes [7], namely cohesin-dependent [8] and chromatin state-driven mechanisms, which give rise to partially overlapping structures and act antagonistically in certain contexts [9–13]. In this review, we discuss cohesin-independent modes of genome organisation, with particular emphasis on epigenetic state-driven contacts. We also consider the links between epigenome-mediated genome folding and gene expression control. Finally, we discuss historical and recent work on whether and how these regulatory mechanisms could be transmitted through cell division to contribute to the mitotic memory of gene expression states.

## Relationship between genome folding and the epigenome

The organisation of chromatin is tightly linked to its epigenetic state. Two major chromatin types make up the genome, euchromatin (see Glossary) and heterochromatin. Euchromatin encompasses genomic regions with low chromatin density, active histone modifications and active genes, whereas heterochromatin corresponds to transcriptionally silent regions with a repressive histone landscape and higher chromatin density. Heterochromatin can be further divided to three subtypes: (i) constitutive heterochromatin enriched in histone H3 lysine 9 di- and trimethylation (H3K9me2/3); (ii) facultative heterochromatin corresponding to regions enriched in H3K27me3; and (iii) quiescent chromatin regions, which are not enriched in specific marks or components and are generally not expressed [14]. Active euchromatin and inactive heterochromatin are spatially segregated in the nucleus in a fashion that is dictated by both their homotypic attraction [13,15–21] and their association with specific subnuclear structures [22–27].

## Euchromatin and heterochromatin occupy distinct nuclear positions

**Figure 1:**
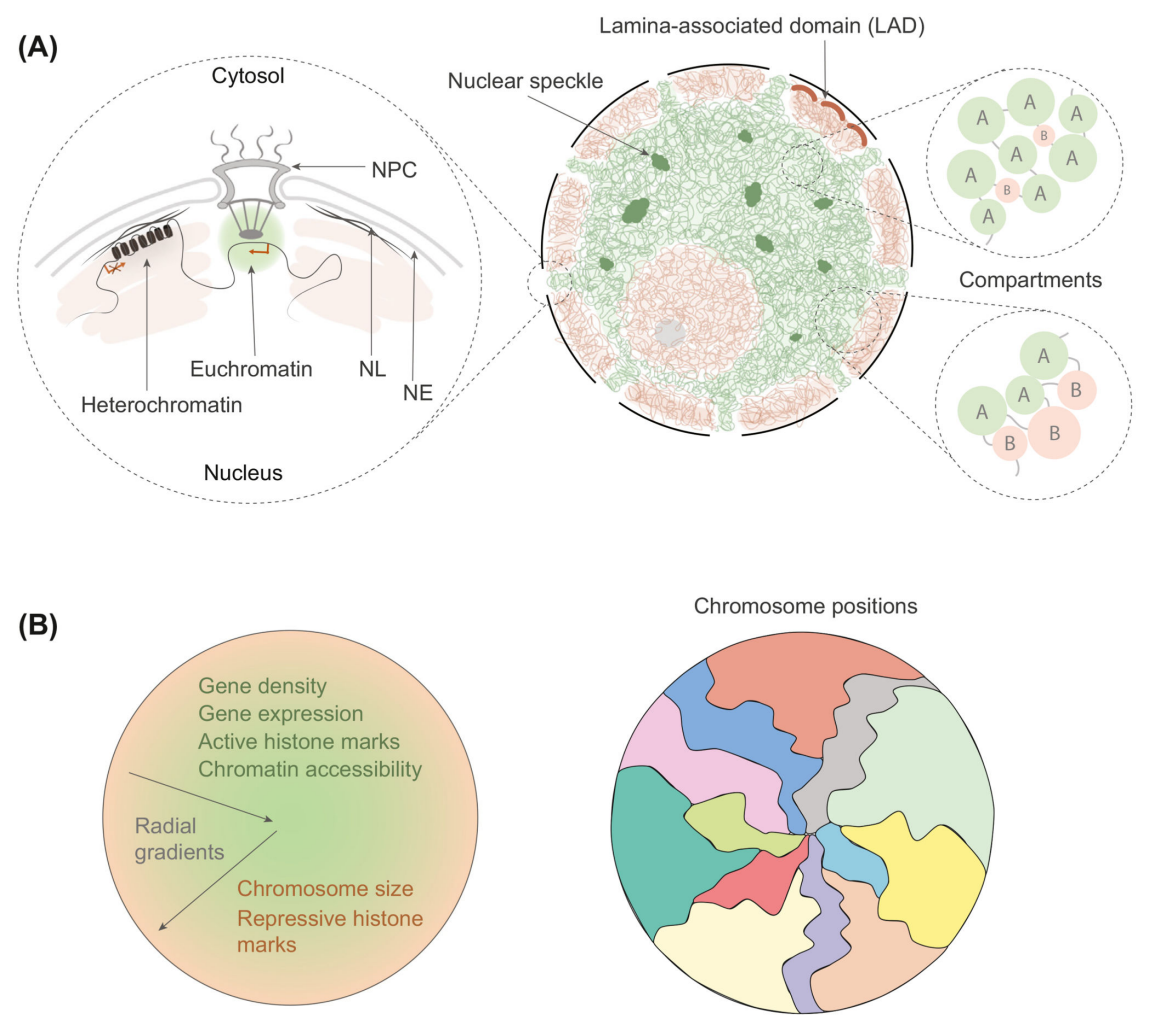
Global epigenome-mediated genome organisation. (A) Heterochromatin localises to the nuclear and nucleolar peripheries, whereas euchromatin resides in the nuclear interior. Heterochromatin is excluded from the vicinity of nuclear pores, which constitute a chromatin environment permissive for transcription. (B) Chromatin features organised along radial gradients (left) and chromosome territories in the nucleus (right). Repressive chromatin signatures and large chromosomes tend to show a more peripheral localisation, while active chromatin signatures and gene density increase toward the nuclear interior. Abbreviations: NE, nuclear envelope; NL, nuclear lamina; NPC, nuclear pore complex.

Electron microscopy studies during the 1950s revealed that chromatin had a distinct appearance in different regions of the nucleus. Notably, heterochromatin shows strong association with the nuclear periphery (Figure 1A), which is conferred by interactions with the nuclear lamina and its associated proteins [22], and the periphery of nucleoli. Accordingly, the nuclear lamina and nucleoli are considered hubs for the organisation and regulation of repressive genomic domains with overlapping functions. By contrast, euchromatin resides in the nuclear interior and in the vicinity of nuclear pore complexes (NPCs), where the nucleoporin TPR counteracts the peripheral localisation of heterochromatin [28,29] (Figure 1A). Using genomic loci positioning by sequencing (GPSeq), a study mapped the radial positions of genomic loci and integrated them with the linear distribution of histone modifications and epigenetic state. This confirmed a global organisational principle where active chromatin marks are arranged along continuous radial gradients increasing from the nuclear periphery to the nuclear interior, while repressive chromatin marks show the opposite trend [30,31] (Figure 1B). Radial nuclear organisation also governs the positioning of chromosomes, where large and/or gene-poor chromosomes preferentially localise to the nuclear periphery [32–34]. Of note, chromosome size alone is not an accurate predictor of radiality, because chromosome positioning is equally influenced by gene density and expression, as well as by GC content [30] (Figure 1B). The functional significance of radial nuclear organisation remains elusive, but it is thought to be involved in the spatial sequestration of genomic functions, such as DNA repair pathway choice [35] or splicing outcome [36].

## Chromatin compartments

The spatial segregation of euchromatin and heterochromatin has been equally observed by orthogonal sequencing-based approaches that gave rise to the widely used two-state chromatin compartment model [37,38] (Figure 1A). In pairwise interactions maps (such as those produced by Hi-C), preferential homotypic interactions of different chromatin types appear as characteristic alternating contact patterns (‘checkerboard’ or ‘plaid’) spanning large genomic distances. Compartment interactions observed by Hi-C and related techniques correlate with histone modification and chromatin accessibility landscapes: while A compartments are observed in regions of overall open chromatin with active genes and activating histone marks, B compartments are associated with chromatin domains that are, in general, closed and repressed [37,38]. Accordingly, A and B compartments are also positioned radially, commonly associating with the nuclear interior and periphery, respectively [30,31], with lamina-associated domains (LADs) showing strong correlation with B compartment identity.

While compartments are frequently considered as multi-megabase structures, ultra-deep Hi-C maps revealed that compartmentalisation is equally present at much finer scales [39,40]. Namely, active regulatory elements, such as enhancers and promoters, nearly always localise to A compartments, even when flanking regions do not. Moreover, certain genes, especially long genes with paused polymerase, can show discordant localisation, where the transcription start and termination sites belong to different compartments. This indicates that subgenic genome organisation precisely follows the distribution of activating histone marks, which in turn is tightly linked to chromatin compartmentalisation even at the kilobase scale. However, this fine-scale compartmentalisation is hard to reconcile with the spatial segregation of compartments at opposing locations within the cell nucleus, suggesting that alternate chromatin compartments must not only constitute large domains, but also finer nanodomains interspersed within the nuclear space (Figure 1A).

Although the molecular factors that mediate chromatin compartmentalisation differ between chromatin types, compartmentalisation is generally thought to be achieved through redundant phase separation-like interactions of epigenetically similar chromatin regions. While such chromatin organisation may be partially intrinsic [13,15], proteins that associate with histone modifications and act as bridging factors have a key role in this process. In the case of the B compartment, heterochromatin protein 1 (HP1) binding to methylated H3K9 segregates constitutive heterochromatin [17,18], while Polycomb repressive complexes (PRCs) sequester facultative H3K27me3 heterochromatin [19–21]. Driving forces of compartmentalisation in the A compartment are less well understood. *In vitro*, acetylated chromatin only phase separates in the presence of the bromodomain protein Brd4 [15], while in embryonic stem cells Brd2 is thought to play a key role [16]. However, a recent preprint reached contradictory conclusions by finding Brd2 dispensable for compartmentalisation [41]. In addition, various factors involved in gene expression regulation, transcription and splicing are thought to be involved in the partitioning of active chromatin through the formation of nuclear condensates [23–27]. Genome compartmentalisation is further reinforced by association with nuclear locales, such as nuclear speckles in case of A chromatin, or the nuclear lamina and nucleoli for B chromatin. By contrast, compartment segregation is counteracted by cohesin-mediated loop extrusion [11,13,16,42] and condensin-driven chromosome condensation [13]. Importantly, the behaviour of the two compartments is interlinked, because biological conditions that lead to global chromatin opening and activation also lead to fortified B compartment contacts [25,43–45]. This indicates that the two compartments exist in an equilibrium, where reinforcing the chromatin state of one can drive the enhanced segregation of the other.

### Chromatin composition-driven genome organisation in gene expression regulation

In agreement with its close link to chromatin state, genome folding can be modulated as epigenetic changes occur in response to developmental and environmental cues. Accordingly, a rewiring is often observed during development and pathogenesis at all organisational layers of the 3D genome. The causal relationship between changes in chromatin organisation and gene expression states has been difficult to disentangle, but increasing evidence suggests that the function of genome folding can be partially uncoupled from other regulatory mechanisms. This led to a global view where 3D genome organisation has at least a partially causative role in gene expression control, but the extent to which this occurs is highly locus and cellular context dependent.

### Epigenome-driven global genome folding reinforces transcriptional states

A plethora of studies highlighted concomitant changes between gene expression state and 3D genome organisation, in terms of both nuclear positions [46–48] and chromatin compartmentalisation. Changes in nuclear positioning during development can range from individual loci and/or compartments to entire chromosomes, where, in general, gene activation is associated with a more internal positioning to the nucleus. Although gene repositioning to the periphery can attenuate gene expression via contacts with the nuclear lamina [49–51], some genes in peripheral chromatin domains (LADs) escape transcriptional repression [52]. Conversely, gene dissociation from the nuclear periphery is not always accompanied by gene activation [53], indicating that nuclear positions are not necessarily sufficient to drive transcriptional repression, but reinforce regulatory states instead.

Compartment changes between different cellular contexts are widespread, with only ~40% of the human genome maintaining stable compartment identity across different cell types [54]. Compartment changes are well correlated with transcriptional changes that occur during cellular state transitions [55–58], but cause–consequence relationships vary according to genomic position and biological condition. For example, a major transcriptional response can take place without changes in chromatin compartments during heat shock and, vice versa, tethering genomic regions to different nuclear subcompartments does not necessarily drive gene expression changes [59,60]. Studies that looked at the temporal relationship between gene expression and compartment changes reported their close coupling during time-course experiments [45,56,61]. However, while compartment changes preceded gene activation in certain cases [56], the inverse was true in others [45], indicating a highly context-dependent biological role of compartments in gene expression control. It is generally thought that genes in the A compartment are more responsive to external and internal cues, whereas the B compartment serves to provide a more stable, repressive state. However, the extent to which compartments have direct, biological roles and how much they form as a consequence of genome function remain to be understood.

### Epigenetic state-driven *cis* contacts in gene expression control

**Figure 2:**
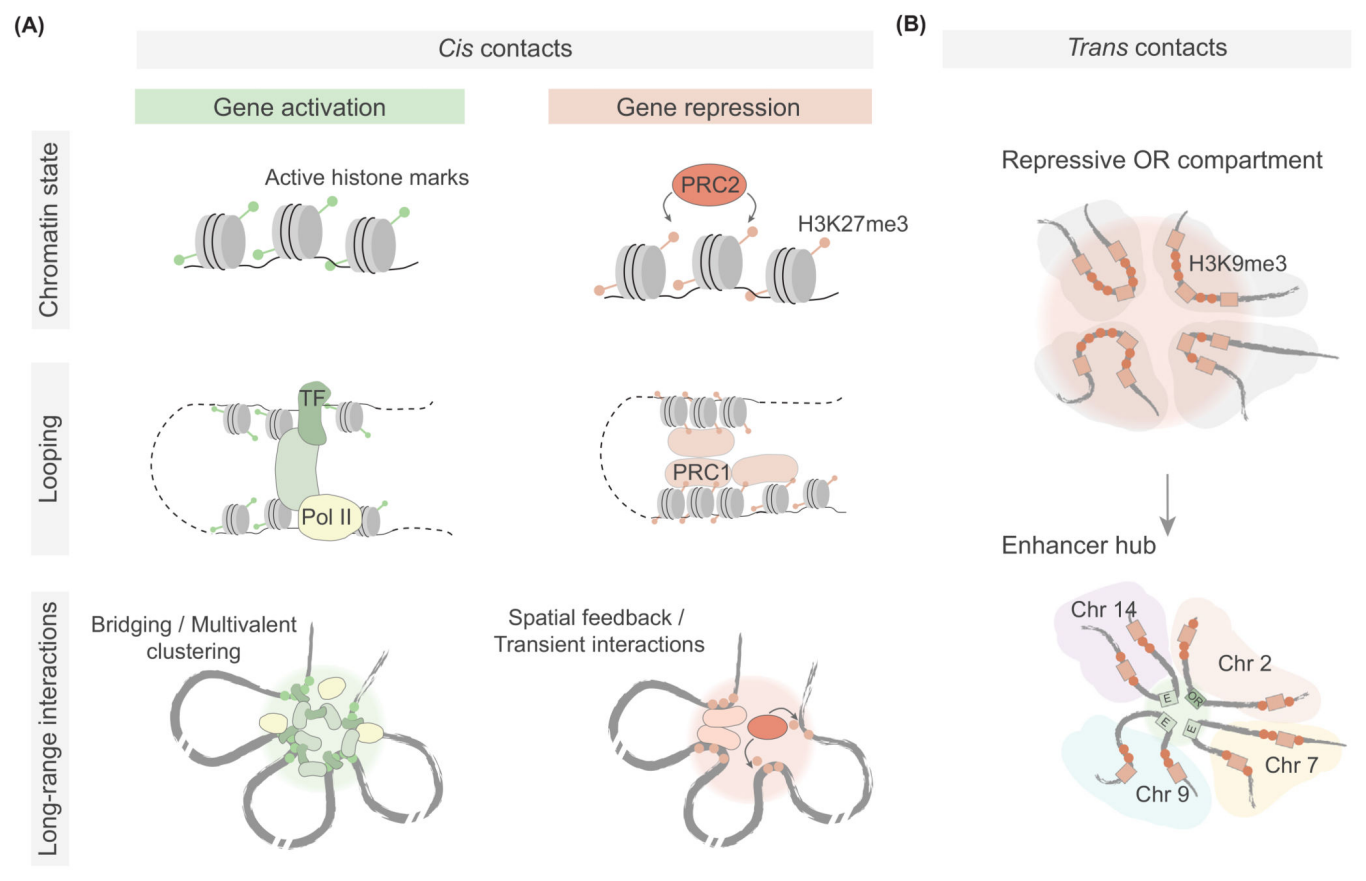
Epigenetic state-driven contacts in gene regulation. (A) Epigenetic state-driven *cis* contacts mediating gene activation (left panels) and gene repression (right panels), and histone modification landscapes reflecting transcriptional states (top panels). Upon gene activation, looping factors establish physical contact between transcription factors and transcription machinery. At Polycomb loci, Polycomb-repressive complex (PRC)-2-deposited H3K27me3 recruits PRC1 complexes, which drive local compaction and chromatin looping (middle panels). Higher-order and long-rage organisation (lower panels) can involve bridging and/or multivalent interactions that induce spatial clustering and feedback. (B) In mouse olfactory sensory neurons, the olfactory receptor (OR) gene compartment is mediated by intrachromosomal heterochromatin interactions (top panel), which eventually leads to the formation of a *trans*-acting enhancer hub that activates the expression of a single OR gene.

Besides the global sequestration of active and inactive chromatin regions, epigenetic states can equally drive focal genomic contacts via chromatin looping between *cis* regulatory elements and their promoter targets. Although chromatin looping is often attributed to the activity of the cohesin complex (Box 1) and its interaction with the architectural protein CTCF, loops can form independently from it. These loop extrusion-independent loops mediated by the epigenetic machinery can occur over various distances, from a few kilobases to several megabases, in both activating and repressive contexts (Figure 2A).

A widely accepted concept is that distal *cis* regulatory elements, or enhancers, drive gene expression from promoters partially through physical contacts mediated by chromatin looping [62,63]. Although the simplicity of this model has been questioned by conflicting observations that point out high context dependency [64–67], interaction maps overall feature a strong correlation between the level of gene expression and enhancer–promoter (E–P) contact frequency [68]. High-resolution studies demonstrated that fine-scale regulatory contacts that occur between enhancers and promoters are largely independent from the action of cohesin and CTCF [68–71]. These observations were made using degron cell lines, which found that E–P contacts are often maintained upon the acute depletion of components and modulators of the loop extrusion machinery [72]. The conclusion that *cis* regulatory contacts are loop-extrusion independent is supported by the fact that, while acute depletion of cohesin and its interactors leads to major changes in submegabase-scale genome organisation, it only causes modest changes in transcription [69]. Recent analysis suggests that cohesin-mediated loop extrusion is only required for regulatory E–P contacts when enhancers are located at large distances from promoters, possibly reconciling these apparently conflicting results [73,74]. Instead, E–P contacts appear to be linked to the presence of a functional transcription machinery, because depletion of RNA Polymerase II (RNAPII) weakened E–P interactions [12]. Interestingly, RNAPII depletion also led to the engagement of new CTCF anchors. This indicates not only that cohesin/CTCF antagonise epigenetic contacts, but, vice versa, chromatin state-driven interactions also restrict CTCF-dependent anchoring of cohesin loops [12]. In addition, the transition from the initiation to the elongation state of RNAPII was found to be linked to cell type-specific loop formation during cell differentiation [75], highlighting an intricate interplay between E–P looping and the activity state of the transcriptional machinery [76].

The molecular factors and mechanisms that confer regulatory connectivity between *cis* elements remain unclear. Various transcription factors and chromatin-associated proteins have been implicated in bringing distal loci into proximity, but few have been shown to unequivocally mediate chromatin looping. A promising candidate for such role was the Mediator complex [77], which was implicated in physically bridging enhancers and promoters to stimulate transcription [78,79]. Subsequent studies uncoupled the function of Mediator in transcription from chromatin looping, questioning its role as a looping factor [80–84]. However, independent work confirmed that Mediator can favour E–P contacts in the presence of cohesin [85], warranting mechanistic work to examine the exact function of Mediator in chromatin folding. The ubiquitous transcription factor YY1 has also been proposed to mediate E–P contacts [86]; however, further evidence using degron cell lines disproved such a universal role for YY1 in E–P looping [69]. Instead of a universal component, evidence points to the existence of cell type-specific looping factors that convey regulatory specificity in different biological conditions. For example, in erythroblast cells, the transcription cofactor LDB1 spatially clusters tissue-specific transcription factors [87–89], in order to induce transcription through the formation of chromatin loops.

An important feature of epigenetic state-mediated interactions is that they can form over extremely long genomic distances. For long-range (>400 kb) enhancer activity, a preprint highlighted the requirement of a novel conserved *cis*-acting element (range extender or REX) at certain developmental loci [90]. At the multi-megabase scale, a study featured the universal presence of ultra-long-range interactions between active chromatin regions [91] that form in a variety of conditions, cell types and organisms. Although interaction strength over long distances is linked to the level of transcription and H3K27 acetylation, it is independent of individual transcription factors, chromatin-associated proteins (BRD4, Mediator, and EP300), cohesin/CTCF, Polycomb, or transcription itself. Instead, molecular simulations revealed that such organisation could be achieved by the nonspecific affinity of multivalent binding factors at active chromatin sites. This suggests that mechanisms related to those that dictate higher-order nuclear organisation equally drive the formation of affinity-based focal genomic contacts.

3D chromatin contacts have also been extensively implicated in the context of gene repression. The developmental regulator complexes PRC1 and PRC2 sequester their targets in 3D space, which contributes to their repressive function. The Polycomb spatial network is thought to provide a regulatory topology that keeps genes and their enhancers in a silenced but poised state [92–95]. In addition, clustering of Polycomb targets allows the long-range spreading of H3K27me3, as well as spatial feedback through transient contacts that contribute to the propagation of a repressive epigenetic state [96,97]. Furthermore, in addition to establishing repressive long-range contacts, Polycomb components can be associated with active gene loops [98–102]. The precise chromatin changes and mechanisms that can turn repressive contacts into active loops are not known and require future research. Polycomb interactions are strengthened upon cohesin removal, again highlighting an antagonistic relationship between cohesin-mediated and epigenetic state-driven structures [9]. Recent evidence suggests that, in mouse embryonic stem cells, loci enriched in H3K9me3 also form focal contacts under certain conditions, and that these looping events correlate with gene expression downregulation [45]. Thus, while chromatin looping is often discussed in the context of E–P loops that positively regulate gene expression, it has an equally prominent role in gene silencing.

### *Trans* regulatory contacts

Besides looping of sequences on the same chromosome, regulatory contacts can also form in *trans*, between different chromosomes. These regulatory *trans* contacts are rare and have been characterised only in a few biological contexts. A well-understood example of how interchromosomal contacts can regulate gene expression comes from mouse olfactory sensory neurons that each express one out of ~2000 olfactory receptor (OR) genes located on 18 different chromosomes (Figure 2B). Through a mechanism driven by interactions of heterochromatin, the transcription factor LHX2, and the adapter protein LDB1, OR genes aggregate into a repressive compartment that prevents multigenic transcription [103]. Subsequently, the formation of a multichromosomal enhancer hub leads to the activation of a single gene that is stochastically chosen for expression by an ensemble of feedback mechanisms [104–106]. A *trans*-acting, cell type-specific enhancer has also been implicated in the positive regulation of Tead4 expression in mouse trophoblast stem cells [107], and interchromosomal contacts were found to trigger epigenetic inheritance of H3K27me3 in *Drosophila* [108]. The sparsity of *trans* contacts makes it challenging to study them, but these recent advances point to the fact that *trans* contact might be more prevalent in gene regulation than previously thought.

## Cellular memory and the 3D genome

### Mitotic changes in the nucleus

**Figure 3:**
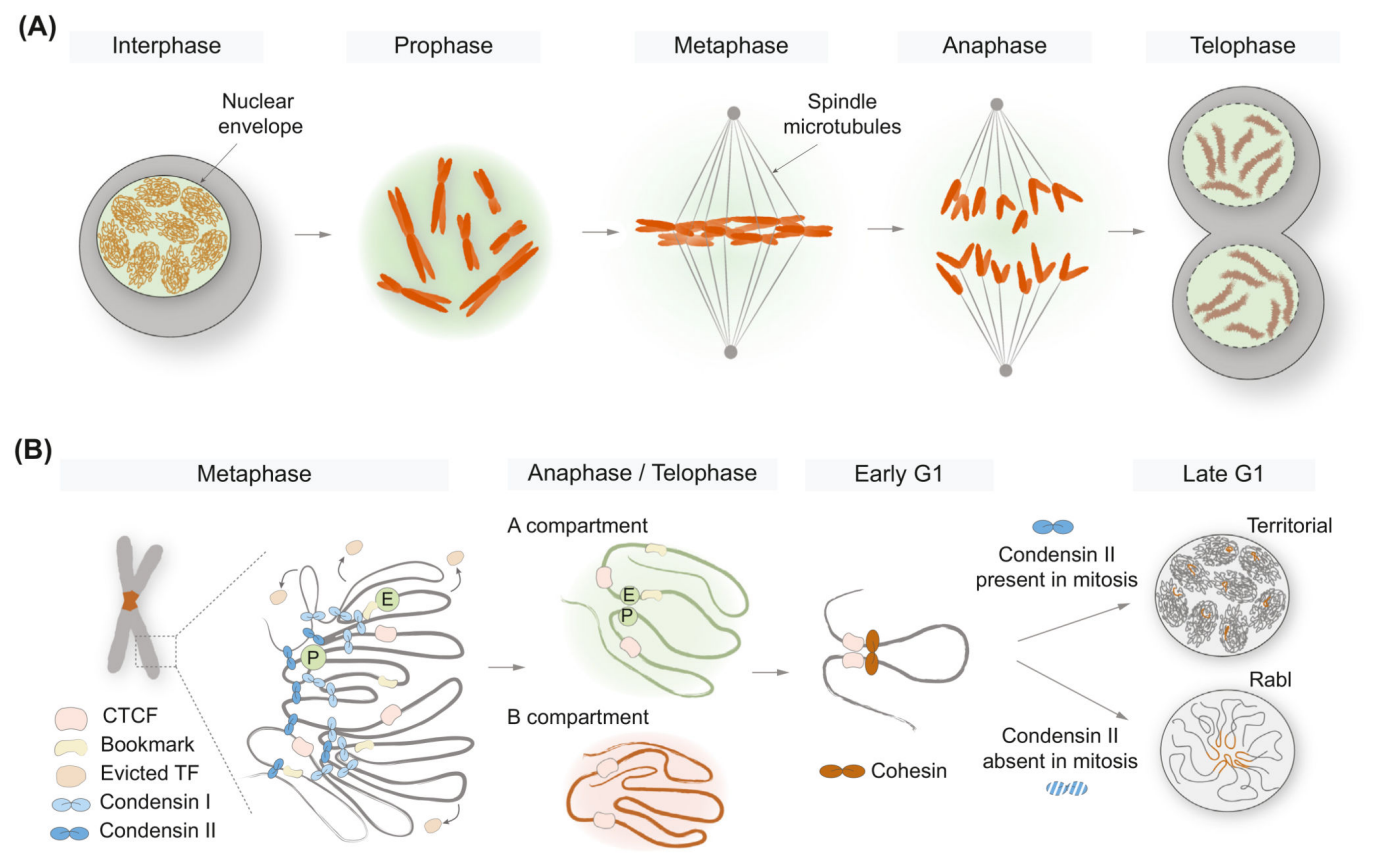
Chromosome conformation changes during the cell cycle. (A) When cells divide, chromosomes condense, and the nuclear envelope disassembles in prophase. Chromosomes align on the metaphase plate and are captured by spindle microtubules in metaphase. In anaphase, chromosomes segregate to opposite spindle poles. Mitosis ends with telophase, where chromosomes decondense and the nuclear envelope reforms. (B) Metaphase chromosomes are organised into consecutive loop arrays by Condensin I and II. Most transcription factors (TFs) are evicted from mitotic chromosomes, but bookmarking factors are retained. At anaphase/telophase, condensin dissociates from chromosomes, which allows enhancer (E)–promoter (P) loops and A/B compartments to progressively reform. Although CTCF binding is retained to some extent, cohesin only associates with chromosomes during early G1. Finally, global organisation of the interphase nucleus depends on its previous mitotic compactions state: chromosomes compacted by Condensin I and II form territorial nuclei, while those compacted in the absence of Condensin II arrange in a Rabl-like configuration with centromeres clustering together.

Chromatin states must be accurately maintained upon cell proliferation to preserve cellular identity. This occurs through cell division, which is divided into well-defined, temporally separated stages (Table 1): the duplication of genetic material (S phase); its subsequent partitioning into daughter cells (M phase); and the intervening gap phases (G1 and G2). Mitosis, the process of nuclear division, can be further divided into five major stages (Figure 3A). Mitosis starts with prophase, where interphase chromatin organisation, including chromatin loops and interaction domains, is removed by chromosome condensation. Concomitantly, chromosomes are rearranged into consecutive loop arrays through the action of condensin complexes [8,109]. Compartment contacts are equally lost, which is directly due to the activity of condensin, because interphase-like compartments gradually reform on condensin-depleted mitotic chromosomes [13]. In parallel, in higher eukaryotes, the nuclear envelope and lamina disassemble in prophase [110], leading to the interruption of lamina–chromatin contacts. Condensed chromosomes are then captured by spindle microtubules in metaphase and become segregated in anaphase. A more peripheral localisation of chromosomes in interphase is linked to higher mitotic segregation errors, indicating that nuclear organisation may have implications for the occurrence of aneuploidies and, thus, in the broader sense, for genome evolution [111].

### Re-establishment of an interphase nucleus

Mitosis ends with telophase, where the effects of prophase are reversed and genome architecture reforms in a sequential manner [112] (Figure 3B and Table 1). In telophase, condensing dissociates from chromosomes, which results in the loss of mitotic loops. Given that cohesin is slower to reassociate with chromosomes, a transient condensin- and cohesin-free folding intermediate forms [113]. The reassembly of the nuclear envelope also starts on the surface of decondensing chromosomes [110,114]. Concomitantly with the disassembly of mitotic loops, compartment formation begins, with short-range interactions reappearing as early as anaphase/telophase, followed by their progressive definition and expansion over longer distances [112,113,115–117]. E–P loops and ultra-long-range interactions reform equally early on decondensing chromosomes, before the reformation of cohesin/CTCF-mediated structures [91,112,118]. Instead, the reformation of E–P contacts was found to partially depend on the presence of RNAPII during mitosis and G1 re-entry [119]. These observations suggest that epigenetic state-driven contacts are chromatin-intrinsic features that are not only maintained, but also form independently from the looping activity of cohesin and condensin complexes.

### Cellular memory and genome folding

Mitosis is accompanied by severe, global transcriptional downregulation [120], which is followed by the rapid postmitotic reactivation of selected genes. This is referred to as mitotic bookmarking and was initially attributed to transcription factors that preserve their sequence-specific binding during mitosis [121–123]. It has been demonstrated since that bookmarks also include certain chromatin readers [124], pluripotency factors [125–127], transcription co-activators [128], and even transcriptional repressors [129]. There is increasing evidence that certain chromatin features related to genome organisation are also retained on mitotic chromosomes and can function as bookmarks. Such features include chromatin accessibility itself [130,131], the chromatin remodelling complex SWI/SNF [132], the architectural protein CTCF [133], and histone acetylation at H3K27 [117,134]. Overall, these factors contribute to the preservation of functional chromatin states at bookmarked promoters to support efficient gene reactivation during mitotic exit.

However, functional data can only explain a fraction of the reconstitution of cellular identity in daughter cells [121], indicating the presence of as yet unexplored mechanisms that ensure the stable maintenance of gene expression states across cell generations. The extent to which 3D genome organisation can contribute to the mitotic transmission of functional chromatin states is unclear. While some early studies observed mitotic transmission of radial chromosome positions [135,136], others found stochastic chromosome reshuffling following cell division [114,137]. Later, it was shown that the histone mark H3K9me2 coordinates the positioning of peripheral heterochromatin to the reforming nuclear lamina before mitotic exit [138]. Altogether, although poorly understood, such evidence indicates the existence of nuclear constituents that act as architectural guideposts to reconstitute the organisation of interphase nuclei.

It is becoming increasingly appreciated that, despite the apparent lack of regulatory structures in mitosis, some of the interphase folding programme is transmitted through mitosis in a chromosome-intrinsic manner. For example, a recent preprint showed that compartment segregation is inherited via mitotic chromosomes, as are interactions that form between bookmarked and cell type-specific *cis* regulatory elements [139]. Although not visible in contact maps, imaging-based research reported that LADs remain spatially segregated from active chromatin stretches even in prometaphase and metaphase, after nuclear envelope disassembly [114]. Chromosome-intrinsic compartment segregation has also been detected on condensin-depleted mitotic chromosomes, where long-range compartment contacts were found to form in the absence of accessory proteins, such as HP1, which are normally thought to have a key role in shaping interphase architecture [13].

In interphase, simulations demonstrated that organisation of chromosomes depends on their prior mitotic conformation [140], indicating that chromosome folding can carry information about the history of the cell. This is supported by observations *in vivo*, because global interphase genome folding was found to depend on the condensin complex that carried out mitotic chromosome condensation in the previous cell cycle [141] (Figure 3B). On a finer scale, transient epigenome perturbation showed that changes in genome conformation can outlast those in the histone modification landscape, and that these could be linked to prolonged changes in gene expression [45]. The potential of genome folding to carry memory might be explained by hysteresis, a newly emerging principle in 3D genome organisation. Indeed, hysteresis was found to be critical to model certain characteristics of genome folding, ranging from *cis* contacts in gene expression control [142] to the organisation of the interphase nucleus [140]. Orthogonal biophysical modelling studies have equally shown that 3D genome folding might be a critical element to promote long-range spreading of epigenetic signal and stabilise epigenetic memory in interphase cells [143–146]. Such evidence provides further support to the association between 3D genome folding and cellular memory.

## Concluding remarks

Epigenetic state and 3D genome architecture are intimately linked at most organisational layers, including nuclear positioning, compartment segregation, interchromosomal interactions, as well as short- and long-range intrachromosomal contacts. Given that it has been shown to contribute to gene expression regulation, chromatin folding is widely considered as part of the epigenome. An important feature of the epigenome is the ability to convert short-lived signals to long-lived changes in gene expression, a concept commonly referred to as epigenetic or cellular memory [147]. Due to the complete elimination of interphase chromosome structures in mitosis, it has been questioned whether regulatory chromatin contacts could contribute to bookmarking and, more broadly, to cellular memory (see Outstanding questions). Although mounting evidence suggests that certain 3D genome features have such capacity, it will be critical to address this question using experimental approaches that uncouple gene regulation from architectural changes. One such strategy is to perform time-series analyses of changes in cellular states following transient events. This approach has been successfully used to assess which epigenetic and/or cellular features can be reversed following short-lived epigenome perturbations [45,148], shedding light on principles of chromatinbased memory. Mechanistically, there is a pressing need for molecular tools that can uncouple effects on the linear chromatin landscape from 3D contacts. Insulator sequences [101,149] or the use of mutants that interfere with spatial clustering, but leave enzymatic and chromatin binding activities intact [84], will be essential to dissect cause–consequence relationships between chromosome folding, histone landscape, and gene expression.

Due the extreme restructuring of nuclear content during mitosis, cell division can serve as a key decision point to either maintain or modify cellular states. Similarly to other epigenomic features, individual cells exhibit significant cell-to-cell variability in their 3D architectures (Box 2), which may be linked to transcriptional fluctuations and heterogeneity. In other instances, transient contacts can trigger stable gene expression changes, but how these transient signals are converted to stable regulatory information remains to be understood. Unlike other carriers of epigenetic information that modulate transcription [150–152], it is unknown if any system ensures the symmetric presence of regulatory 3D contacts in daughter cells. Ever-evolving microscopy and molecular biology methods that combine single cell analyses with lineage history will be critical to assess the extent to which individual daughter cells reproduce parental chromosome conformations. If nuclear organisation has a *bona fide* role in cellular memory, chromatin folding should be transmitted and closely related cells should share architectural features. Another critical cell cycle stage is DNA replication, during which genome architecture is perturbed locally when the replication fork starts and transverses genome replication domains in a manner linked to chromatin folding. Recent work showed how chromatin composition can be inherited through DNA replication [153–155], but how this is reflected in the 3D architecture of chromosomal loci is unknown and requires improvement in current technologies. Moreover, approaches that provide precise information on cell cycle stage could uncover whether architectural differences between sister cells decrease or increase with time passed since the last mitosis and/or DNA duplication event. Such approaches will be critical to understand how genome replication and mitotic events might be used in cell fate decisions to modulate or maintain cellular identity, as well as which molecular factors contribute to this process.

## Figures and Tables

**Table 1:** Nuclear and chromosomal events during and following mitosis

Cell cycle stage	Cytological features	Nuclear integrity	Genome folding
Prophase	Chromosome condensed by condensin; centrioles move apart	Nuclear envelope breakdown	Mitotic chromatin loops [109], spatial segregation of LADs [114], chromosome individualisation
Metaphase	Chromosomes line up at equator and are captured by spindle microtubules	Mixing of nucleoplasm with cytoplasm	
Anaphase	Sister chromatid cohesion is removed, chromosomes move apart	Nuclear envelope reassembly starts	Condensin-dependent loops removed [113]; short-range compartment interactions [112], E-P loops [112,113,139], ultra-long-range contacts [91] emerge; contact domain formation starts [112], H3K9me2 positioned to nuclear lamina [138]
Telophase	Chromosomes are at spindle poles, decondensation begins	Nuclear envelope reassembled	
G1	Daughter cells separated	Intact daughter nuclei	Long-range compartment interactions [112,113,170], cohesin loops, TADs [112,113], transient E-P loops removed [112]

## Box 1 Genome folding by loop extrusion

Structural maintenance of chromosome (SMC) complexes are ring-shaped motor protein complexes with critical roles in genome function, ranging from mitotic chromosome segregation to DNA recombination and repair [8]. SMC complexes have received much attention due to their active role in structuring interphase and mitotic chromosomes. According to the current model, cohesin and condensin mediate non-topological chromosomal interactions via loop extrusion: the SMC complex binds two adjacent DNA sites; then, using its enzymatic activity, it reels the DNA inside the SMC ring, moving one or both contact points away from the other. This leads to the bridging of more distant sites and the progressive enlargement of the chromatin loop until an extrusion barrier is encountered. In mammalian cells, loop extrusion activity of cohesin is often halted at convergently oriented CTCF sites, giving rise to topologically associating domains (TADs) and cohesin loop domains. The visionary idea of loop extrusion dates back to 2001 [156], with initial evidence coming only a decade later from *in silico* models that indicated that loop extrusion can explain key aspects of chromatin loop and domain formation [157–159]. Recent work provides experimental support for the loop extrusion model: condensin [160] and later cohesin [161,162] complexes were observed to extrude naked DNA loops *in vitro*. It remains unclear how SMC complexes behave when they encounter native chromatin substrates, but they appear to be able to compact nucleosome-bound DNA [163] and traverse roadblocks larger than their ring size [163], indicating that chromatin-related factors and DNA processes could be accommodated by loop-extruding factors *in vivo*.

## Box 2 Cell–cell variability in genome architecture

Much of our understanding of how genomes are folded in 3D space comes from proximity ligation methods, which offer detailed, genome-wide information on pairwise contact probabilities in cell populations. Microscopy and sequencing-based single cell methods have been critical in revealing that genome folding exhibits significant cell-to-cell heterogeneity at all organisational layers. This raised some of the key current questions in 3D genome field, such as the biological significance of variability between cells, or the presence of chromosomal structures observed in contact maps in individual cells. Studies addressing genome folding at the single cell level revealed that, despite the substantial heterogeneity, chromosome territories, A/B compartments, TADs, Polycomb domains, chromatin loops, as well as chromosomal domains organised around various nuclear bodies, form in individual cells [97,164–169]. However, such methods along with live-cell imaging studies uncovered that focal interactions, including chromatin loops and E–P contacts, are dynamic structures that are only present in a fraction of cells at a given time [62,169]. Conversely, super-resolution imaging studies highlighted the presence of smaller chromatin nanodomains (CNDs) inside TADs, which form in a chromatin state-dependent manner at variable genomic positions [165].

Together, these reports indicate that, although genome-folding principles uncovered by population-based and single cell methods are highly consistent, certain architectural features remain inaccessible to population-based methods due to their high degree of variability, while other features may appear more accentuated than their actual prevalence among individual cells. Whether cell–cell variability in genome architecture is linked to transcriptional heterogeneity or has other biological functions remains to be determined.

## Outstanding questions

What are the global rules governing cause–consequence relationships between gene expression changes and changes in genome folding?

To what extent does 3D genome folding have a regulatory role in gene expression control?

How are transient 3D chromatin contacts converted to stable gene expression signals?

Can 3D chromatin contacts be transmitted to daughter cells?

What are the molecular factors that provide architecture-based memory?

What is the role of mitosis in cellular fate decisions?

## Glossary

Chromatin accessibility: physical access to DNA that scales with chromatin activity.
Chromosome territories: regions of the nucleus that are preferentially occupied by individual chromosomes.
Degron cell lines: cell lines that enable conditional protein regulation via the addition of a degrader compound, which induces acute degradation of proteins of interest.
Enhancer: distal *cis* regulatory elements that positively regulate gene expression in a tissue-specific manner.
Euchromatin: lightly packed, accessible chromatin that is transcriptionally active in general.
Heterochromatin: electron-dense, compact chromatin that is generally gene poor or silent.
Heterochromatin protein 1 (HP1): structural component of constitutive heterochromatin that exists in different isoforms (HP1α, HP1β, and HP1γ), with both redundant and unique functions.
Histone modifications: histone octamers can be post-translationally modified at different subunits and positions that directly affect gene expression; also influence other DNA-related processes, such as replication, recombination, and repair.
Hysteresis: dependence of the behaviour of a system on its own history.
Mediator: fundamental transcriptional coactivator that constitutes a functional bridge between transcription factors and the transcription machinery.
Nuclear lamina: continuous meshwork of lamins and lamin-associated proteins that lines the nucleoplasmic side of the nuclear envelope.
Nuclear pore complexes (NPCs): macromolecular protein complexes that span the nuclear envelope and ensure nucleocytoplasmic transport.
Polycomb repressive complex (PRC) 1 and 2: critical developmental repressor complexes the deregulation of which is associated with diverse phenotypes in development and disease.
Promoter: DNA sequence to which RNA polymerase binds to initiate transcription of the associated gene.
Proximity ligation methods: techniques (e.g., Hi-C and Micro-C) that convert physical contacts between loci to chimeric DNA molecules to produce genomic interaction maps.
Quiescent chromatin: genomic regions largely devoid of histone modifications, epigenetic signals, and transcriptional activity.

## Highlights

Epigenetic state dictates nuclear organisation, global genome folding, and certain types of focal chromatin contact.

Chromatin state-driven genome folding is often counteracted by cohesin- and condensin-mediated loop extrusion.

3D organisation of the epigenome is tightly linked to cellular identity and provides an additional regulatory layer to safeguard transcriptional states.

Mitosis eliminates all apparent aspects of interphase chromosome organisation, but the epigenetic folding programme is transmitted to daughter cells in a chromosome-intrinsic manner.

Genome folding partially depends on its past state, suggesting that 3D genome organisation contributes to cellular memory.

## References

[1] Flemming W (1878). Contributions to the knowledge of the cell and its vital processes. Arch Mikrosk Anat.

[2] Ptashne M (1986). Gene regulation by proteins acting nearby and at a distance. Nature.

[3] Carter D (2002). Long-range chromatin regulatory interactions in vivo. Nat Genet.

[4] Tolhuis B (2002). Looping and interaction between hypersensitive sites in the active β-globin locus. Mol Cell.

[5] Jerkovic I, Cavalli G (2021). Understanding 3D genome organization by multidisciplinary methods. Nat Rev Mol Cell Biol.

[6] Szalay MF (2024). Evolution and function of chromatin domains across the tree of life. Nat Struct Mol Biol.

[7] Mirny LA (2019). Two major mechanisms of chromosome organization. Curr Opin Cell Biol.

[8] Hoencamp C, Rowland BD (2023). Genome control by SMC complexes. Nat Rev Mol Cell Biol.

[9] Rhodes JDP (2020). Cohesin disrupts polycomb-dependent chromosome interactions in embryonic stem cells. Cell Rep.

[10] Tsang FH (2024). The characteristics of CTCF binding sequences contribute to enhancer blocking activity. Nucleic Acids Res.

[11] Schwarzer W (2017). Two independent modes of chromatin organization revealed by cohesin removal. Nature.

[12] Zhang S (2023). Enhancer–promoter contact formation requires RNAPII and antagonizes loop extrusion. Nat Genet.

[13] Zhao H (2024). Genome folding principles uncovered in condensin-depleted mitotic chromosomes. Nat Genet.

[14] Hoffman MM (2013). Integrative annotation of chromatin elements from ENCODE data. Nucleic Acids Res.

[15] Gibson BA (2019). Organization of chromatin by intrinsic and regulated phase separation. Cell.

[16] Xie L (2022). BRD2 compartmentalizes the accessible genome. Nat Genet.

[17] Sanulli S (2019). HP1 reshapes nucleosome core to promote phase separation of heterochromatin. Nature.

[18] Wang L (2019). Histone modifications regulate chromatin compartmentalization by contributing to a phase separation mechanism. Mol Cell.

[19] Tatavosian R (2019). Nuclear condensates of the Polycomb protein chromobox 2 (CBX2) assemble through phase separation. J Biol Chem.

[20] Plys AJ (2019). Phase separation of polycomb-repressive complex 1 is governed by a charged disordered region of CBX2. Genes Dev.

[21] Ingersoll S (2024). Sparse CBX2 nucleates many Polycomb proteins to promote facultative heterochromatinization of Polycomb target genes. bioRxiv.

[22] Manzo SG (2022). Lamina-associated domains: tethers and looseners. Curr Opin Cell Biol.

[23] Sabari BR (2018). Coactivator condensation at super-enhancers links phase separation and gene control. Science.

[24] Manteiga JC (2019). Pol II phosphorylation regulates a switch between transcriptional and splicing condensates. Nature.

[25] Hu S (2019). Disruption of nuclear speckles reduces chromatin interactions in active compartments. Epigenetics Chromatin.

[26] Sztacho M (2024). The RNA-dependent interactions of phosphatidylinositol 4,5-bisphosphate with intrinsically disordered proteins contribute to nuclear compartmentalization. PLoS Genet.

[27] Lerra L (2024). An RNA-dependent and phase-separated active subnuclear compartment safeguards repressive chromatin domains. Mol Cell.

[28] Schermelleh L (2008). Subdiffraction multicolor imaging of the nuclear periphery with 3D structured illumination microscopy. Science.

[29] Krull S (2010). Protein Tpr is required for establishing nuclear pore-associated zones of heterochromatin exclusion. EMBO J.

[30] Girelli G (2020). GPSeq reveals the radial organization of chromatin in the cell nucleus. Nat Biotechnol.

[31] Bouwman BA (2023). A GC-centered view of 3D genome organization. Curr Opin Genet Dev.

[32] Sun HB (2000). Size-dependent positioning of human chromosomes in interphase nuclei. Biophys J.

[33] Tanabe H (2002). Evolutionary conservation of chromosome territory arrangements in cell nuclei from higher primates. Proc Natl Acad Sci U S A.

[34] Cremer M (2001). Non-random radial higher-order chromatin arrangements in nuclei of diploid human cells. Chromosom Res.

[35] Lemaître C (2014). Nuclear position dictates DNA repair pathway choice. Genes Dev.

[36] Tammer L (2022). Gene architecture directs splicing outcome in separate nuclear spatial regions. Mol Cell.

[37] Hildebrand EM, Dekker J (2020). Mechanisms and functions of chromosome compartmentalization. Trends Biochem Sci.

[38] Li H (2024). Chromosome compartmentalization: causes, changes, consequences, and conundrums. Trends Cell Biol.

[39] Harris HL (2023). Chromatin alternates between A and B compartments at kilobase scale for subgenic organization. Nat Commun.

[40] Goel VY (2023). Region Capture Micro-C reveals coalescence of enhancers and promoters into nested microcompartments. Nat Genet.

[41] Hinojosa-Gonzalez L (2023). Brd2 is dispensable for genome compartmentalization and replication timing. BioRxiv.

[42] Rao SSP (2017). Cohesin loss eliminates all loop domains. Cell.

[43] Michealraj KA (2020). Metabolic regulation of the epigenome drives lethal infantile ependymoma. Cell.

[44] Johnston MJ (2024). TULIPs decorate the three-dimensional genome of PFA ependymoma. Cell.

[45] Paldi F (2024). Transient histone deacetylase inhibition induces cellular memory of gene expression and three-dimensional genome folding. bioRxiv.

[46] Brown KE (1997). Association of transcriptionally silent genes with Ikaros complexes at centromeric heterochromatin. Cell.

[47] Lomvardas S (2006). Interchromosomal interactions and olfactory receptor choice. Cell.

[48] Chambeyron S, Bickmore WA (2004). Chromatin decondensation and nuclear reorganization of the HoxB locus upon induction of transcription. Genes Dev.

[49] Finlan LE (2008). Recruitment to the nuclear periphery can alter expression of genes in human cells. PLoS Genet.

[50] Reddy KL (2008). Transcriptional repression mediated by repositioning of genes to the nuclear lamina. Nature.

[51] Akhtar W (2013). XChromatin position effects assayed by thousands of reporters integrated in parallel. Cell.

[52] Leemans C (2019). Promoter-intrinsic and local chromatin features determine gene repression in LADs. Cell.

[53] Robson MI (2016). Tissue-specific gene repositioning by muscle nuclear membrane proteins enhances repression of critical developmental genes during myogenesis. Mol Cell.

[54] Schmitt AD (2016). A compendium of chromatin contact maps reveals spatially active regions in the human genome. Cell Rep.

[55] Bonev B (2017). Multiscale 3D genome rewiring during mouse neural development. Cell.

[56] Miura H (2019). Single-cell DNA replication profiling identifies spatiotemporal developmental dynamics of chromosome organization. Nat Genet.

[57] Bertero A (2019). Dynamics of genome reorganization during human cardiogenesis reveal an RBM20-dependent splicing factory. Nat Commun.

[58] Zhang K (2020). Analysis of genome architecture during SCNT reveals a role of cohesin in impeding minor ZGA. Mol Cell.

[59] Wijchers PJ (2016). Cause and consequence of tethering a SubTAD to different nuclear compartments. Mol Cell.

[60] Ray J (2019). Chromatin conformation remains stable upon extensive transcriptional changes driven by heat shock. Proc Natl Acad Sci U S A.

[61] Reed KSM (2022). Temporal analysis suggests a reciprocal relationship between 3D chromatin structure and transcription. Cell Rep.

[62] Kawasaki K, Fukaya T (2024). Regulatory landscape of enhancer-mediated transcriptional activation. Trends Cell Biol.

[63] Uyehara CM, Apostolou E (2023). 3D enhancer-promoter interactions and multi-connected hubs: organizational principles and functional roles. Cell Rep.

[64] Winick-Ng W (2021). Cell-type specialization is encoded by specific chromatin topologies. Nature.

[65] Benabdallah NS (2019). Decreased enhancer-promoter proximity accompanying enhancer activation. Mol Cell.

[66] Alexander JM (2019). Live-cell imaging reveals enhancer-dependent sox2 transcription in the absence of enhancer proximity. Elife.

[67] Jerkovic I (2024). A scaffolding element rewires local 3D chromatin architecture during differentiation. bioRxiv.

[68] Hsieh T-HS (2020). Resolving the 3D landscape of transcription-linked mammalian chromatin folding. Mol Cell.

[69] Hsieh THS (2022). Enhancer–promoter interactions and transcription are largely maintained upon acute loss of CTCF, cohesin, WAPL or YY1. Nat Genet.

[70] Aljahani A (2022). Analysis of sub-kilobase chromatin topology reveals nano-scale regulatory interactions with variable dependence on cohesin and CTCF. Nat Commun.

[71] Thiecke MJ (2020). Cohesin-dependent and -independent mechanisms mediate chromosomal contacts between promoters and enhancers. Cell Rep.

[72] de Wit E, Nora EP (2023). New insights into genome folding by loop extrusion from inducible degron technologies. Nat Rev Genet.

[73] Kane L (2022). Cohesin is required for long-range enhancer action at the Shh locus. Nat Struct Mol Biol.

[74] Kim M (2024). Multifaceted roles of cohesin in regulating transcriptional loops. bioRxiv.

[75] Titus KR (2024). Cell-type-specific loops linked to RNA polymerase II elongation in human neural differentiation. Cell Genomics.

[76] Barshad G (2023). RNA polymerase II dynamics shape enhancer-promoter interactions. Nat Genet.

[77] Allen BL, Taatjes DJ (2015). The Mediator complex: a central integrator of transcription. Nat Rev Mol Cell Biol.

[78] Kagey MH (2010). Mediator and cohesin connect gene expression and chromatin architecture. Nature.

[79] Lai F (2013). Activating RNAs associate with Mediator to enhance chromatin architecture and transcription. Nature.

[80] Sun F (2021). The Pol II preinitiation complex (PIC) influences Mediator binding but not promoter-enhancer looping. Genes Dev.

[81] Crump NT (2021). BET inhibition disrupts transcription but retains enhancer-promoter contact. Nat Commun.

[82] Jaeger MG (2020). Selective Mediator dependence of cell-type-specifying transcription. Nat Genet.

[83] El Khattabi L (2019). A pliable mediator acts as a functional rather than an architectural bridge between promoters and enhancers. Cell.

[84] Dimitrova E (2022). Distinct roles for CKM–Mediator in controlling Polycomb-dependent chromosomal interactions and priming genes for induction. Nat Struct Mol Biol.

[85] Ramasamy S (2023). The Mediator complex regulates enhancer-promoter interactions. Nat Struct Mol Biol.

[86] Weintraub AS (2017). YY1 is a structural regulator of enhancer-promoter loops. Cell.

[87] Krivega I, Dean A (2017). LDB1-mediated enhancer looping can be established independent of mediator and cohesin. Nucleic Acids Res.

[88] Liu G (2022). Enhancer looping protein LDB1 regulates hepatocyte gene expression by cooperating with liver transcription factors. Nucleic Acids Res.

[89] Aboreden NG (2025). LDB1 establishes multi-enhancer networks to regulate gene expression. Mol Cell.

[90] Bower G (2024). Conserved cis-acting range extender element mediates extreme long-range enhancer activity in mammals. bioRxiv.

[91] Friman ET (2023). Ultra-long-range interactions between active regulatory elements. Genome Res.

[92] Schoenfelder S (2015). Polycomb repressive complex PRC1 spatially constrains the mouse embryonic stem cell genome. Nat Genet.

[93] Cruz-Molina S (2017). PRC2 facilitates the regulatory topology required for poised enhancer function during pluripotent stem cell differentiation. Cell Stem Cell.

[94] Mas G (2018). Promoter bivalency favors an open chromatin architecture in embryonic stem cells. Nat Genet.

[95] Boyle S (2020). A central role for canonical PRC1 in shaping the 3D nuclear landscape. Genes Dev.

[96] Kraft K (2022). Polycomb-mediated genome architecture enables long-range spreading of H3K27 methylation. Proc Natl Acad Sci U S A.

[97] Murphy SE, Boettiger AN (2024). Polycomb repression of Hox genes involves spatial feedback but not domain compaction or phase transition. Nat Genet.

[98] Kondo T (2014). Polycomb potentiates Meis2 activation in midbrain by mediating interaction of the promoter with a tissue-specific enhancer. Dev Cell.

[99] Loubiere V (2020). Widespread activation of developmental gene expression characterized by PRC1-dependent chromatin looping. Sci Adv.

[100] Zhang Y (2021). The Polycomb protein RING1B enables estrogen-mediated gene expression by promoting enhancer-promoter interaction and R-loop formation. Nucleic Acids Res.

[101] Denaud S (2024). A PRE loop at the dac locus acts as a topological chromatin structure that restricts and specifies enhancer–promoter communication. Nat Struct Mol Biol.

[102] Hanafiah A (2024). PRC1 and CTCF-mediated transition from poised to active chromatin loops drives bivalent gene activation. BioRxiv.

[103] Monahan K (2019). LHX2- and LDB1-mediated trans interactions regulate olfactory receptor choice. Nature.

[104] Markenscoff-Papadimitriou E (2014). Enhancer interaction networks as a means for singular olfactory receptor expression. Cell.

[105] Bashkirova E, Lomvardas S (2019). Olfactory receptor genes make the case for inter-chromosomal interactions. Curr Opin Genet Dev.

[106] Pourmorady AD (2024). RNA-mediated symmetry breaking enables singular olfactory receptor choice. Nature.

[107] Tomikawa J (2020). Exploring trophoblast-specific Tead4 enhancers through chromatin conformation capture assays followed by functional screening. Nucleic Acids Res.

[108] Fitz-James MH (2025). Interchromosomal contacts between regulatory regions trigger stable transgenerational epigenetic inheritance in *Drosophila*. Mol Cell.

[109] Naumova N (2013). Organization of the mitotic chromosome. Science.

[110] Hampoelz B, Baumbach J (2023). Nuclear envelope assembly and dynamics during development. Semin Cell Dev Biol.

[111] Klaasen SJ (2022). Nuclear chromosome locations dictate segregation error frequencies. Nature.

[112] Zhang H (2019). Chromatin structure dynamics during the mitosis-to-G1 phase transition. Nature.

[113] Abramo K (2019). A chromosome folding intermediate at the condensin-to-cohesin transition during telophase. Nat Cell Biol.

[114] Kind J (2013). Single-cell dynamics of genome-nuclear lamina interactions. Cell.

[115] Nagano T (2017). Cell-cycle dynamics of chromosomal organization at single-cell resolution. Nature.

[116] Dileep V (2015). Topologically associating domains and their long-range contacts are established during early G1 coincident with the establishment of the replication-timing program. Genome Res.

[117] Pelham-Webb B (2021). H3K27ac bookmarking promotes rapid post-mitotic activation of the pluripotent stem cell program without impacting 3D chromatin reorganization. Mol Cell.

[118] Zhang H (2021). CTCF and transcription influence chromatin structure re-configuration after mitosis. Nat Commun.

[119] Zhang S (2021). RNA polymerase II is required for spatial chromatin reorganization following exit from mitosis. Sci Adv.

[120] Palozola KC (2017). Mitotic transcription and waves of gene reactivation during mitotic exit. Science.

[121] Palozola KC (2019). A changing paradigm of transcriptional memory propagation through mitosis. Nat Rev Mol Cell Biol.

[122] Ito K, Zaret KS (2022). Maintaining transcriptional specificity through mitosis. Annu Rev Genomics Hum Genet.

[123] Budzynśki MA (2024). A dynamic role for transcription factors in restoring transcription through mitosis. Biochem Soc Trans.

[124] Dey A (2009). Brd4 marks select genes on mitotic chromatin and directs postmitotic transcription. Mol Biol Cell.

[125] Festuccia N (2016). Mitotic binding of Esrrb marks key regulatory regions of the pluripotency network. Nat Cell Biol.

[126] Deluz C (2016). A role for mitotic bookmarking of SOX2 in pluripotency and differentiation. Genes Dev.

[127] Chervova A (2024). Mitotic bookmarking redundancy by nuclear receptors in pluripotent cells. Nat Struct Mol Biol.

[128] Blobel GA (2009). A reconfigured pattern of MLL occupancy within mitotic chromatin promotes rapid transcriptional reactivation following mitotic exit. Mol Cell.

[129] Arora M (2015). RING1A and BMI1 bookmark active genes via ubiquitination of chromatin-associated proteins. Nucleic Acids Res.

[130] Hsiung CCS (2015). Genome accessibility is widely preserved and locally modulated during mitosis. Genome Res.

[131] Teves SS (2016). A dynamic mode of mitotic bookmarking by transcription factors. Elife.

[132] Zhu Z (2023). Mitotic bookmarking by SWI/SNF subunits. Nature.

[133] Chervova A (2023). A gene subset requires CTCF bookmarking during the fast post-mitotic reactivation of mouse ES cells. EMBO Rep.

[134] Liu Y (2017). Widespread mitotic bookmarking by histone marks and transcription factors in pluripotent stem cells. Cell Rep.

[135] Cremer M (2003). Inheritance of gene density-related higher order chromatin arrangements in normal and tumor cell nuclei. J Cell Biol.

[136] Gerlich D (2003). Global chromosome positions are transmitted through mitosis in mammalian cells. Cell.

[137] Thomson I (2004). The radial positioning of chromatin is not inherited through mitosis but is established de novo in early G1. Curr Biol.

[138] Poleshko A (2019). H3k9me2 orchestrates inheritance of spatial positioning of peripheral heterochromatin through mitosis. Elife.

[139] Schooley A (2024). Interphase chromosome conformation is specified by distinct folding programs inherited via mitotic chromosomes or through the cytoplasm. BioRxiv.

[140] Di Stefano M (2021). Polymer modelling unveils the roles of heterochromatin and nucleolar organizing regions in shaping 3D genome organization in *Arabidopsis thaliana*. Nucleic Acids Res.

[141] Hoencamp C (2021). 3D genomics across the tree of life reveals condensin II as a determinant of architecture type. Science.

[142] Xiao J (2021). How subtle changes in 3d structure can create large changes in transcription. Elife.

[143] Owen JA (2023). Design principles of 3D epigenetic memory systems. Science.

[144] Jost D, Vaillant C (2018). Epigenomics in 3D: importance of long-range spreading and specific interactions in epigenomic maintenance. Nucleic Acids Res.

[145] Michieletto D (2016). Polymer model with epigenetic recoloring reveals a pathway for the de novo establishment and 3D organization of chromatin domains. Phys Rev X.

[146] Katava M (2022). Chromatin dynamics controls epigenetic domain formation. Biophys J.

[147] D’Urso A, Brickner JH (2014). Mechanisms of epigenetic memory. Trends Genet.

[148] Parreno V (2024). Transient loss of Polycomb components induces an epigenetic cancer fate. Nature.

[149] Ogiyama Y (2018). Polycomb-dependent chromatin looping contributes to gene silencing during Drosophila development. Mol Cell.

[150] Shipony Z (2014). Dynamic and static maintenance of epigenetic memory in pluripotent and somatic cells. Nature.

[151] Yu C (2018). A mechanism for preventing asymmetric histone segregation onto replicating DNA strands. Science.

[152] Petryk N (2018). MCM2 promotes symmetric inheritance of modified histones during DNA replication. Science.

[153] Wenger A (2023). Symmetric inheritance of parental histones governs epigenome maintenance and embryonic stem cell identity. Nat Genet.

[154] Charlton SJ (2024). The fork protection complex promotes parental histone recycling and epigenetic memory. Cell.

[155] Ostrowski MS (2025). The single-molecule accessibility landscape of newly replicated mammalian chromatin. Cell.

[156] Nasmyth K (2001). Disseminating the genome: joining, resolving, and separating sister chromatids during mitosis and meiosis. Annu Rev Genet.

[157] Alipour E, Marko JF (2012). Self-organization of domain structures by DNA-loop-extruding enzymes. Nucleic Acids Res.

[158] Sanborn AL (2015). Chromatin extrusion explains key features of loop and domain formation in wild-type and engineered genomes. Proc Natl Acad Sci U S A.

[159] Goloborodko A (2016). Chromosome compaction by active loop extrusion. Biophys J.

[160] Ganji M (2018). Real-time imaging of DNA loop extrusion by condensin. Science.

[161] Kim Y (2019). Human cohesin compacts DNA by loop extrusion. Science.

[162] Davidson IF (2019). DNA loop extrusion by human cohesin. Science.

[163] Pradhan B (2022). SMC complexes can traverse physical roadblocks bigger than their ring size. Cell Rep.

[164] Bintu B (2018). Super-resolution chromatin tracing reveals domains and cooperative interactions in single cells. Science.

[165] Szabo Q (2020). Regulation of single-cell genome organization into TADs and chromatin nanodomains. Nat Genet.

[166] Arrastia MV (2022). Single-cell measurement of higher-order 3D genome organization with scSPRITE. Nat Biotechnol.

[167] Cremer T, Cremer C (2001). Chromosome territories, nuclear architecture and gene regulation in mammalian cells. Nat Rev Genet.

[168] Stevens TJ (2017). 3D structures of individual mammalian genomes studied by single-cell Hi-C. Nature.

[169] Gabriele M (2022). Dynamics of CTCF- and cohesin-mediated chromatin looping revealed by live-cell imaging. Science.

[170] Pelham-Webb B (2020). Dynamic 3D chromatin reorganization during establishment and maintenance of pluripotency. Stem Cell Rep.

